# Vessel segmentation for X-ray coronary angiography using ensemble methods with deep learning and filter-based features

**DOI:** 10.1186/s12880-022-00734-4

**Published:** 2022-01-19

**Authors:** Zijun Gao, Lu Wang, Reza Soroushmehr, Alexander Wood, Jonathan Gryak, Brahmajee Nallamothu, Kayvan Najarian

**Affiliations:** 1 Department of Computational Medicine and Bioinformatics, University of Michigan, Ann Arbor, USA; 2 Michigan Institute for Data Science (MIDAS), University of Michigan, Ann Arbor, USA; 3 Department of Internal Medicine, University of Michigan, Ann Arbor, USA; 4 Division of Cardiovascular Diseases, University of Michigan, Ann Arbor, USA; 5 Department of Emergency Medicine, University of Michigan, Ann Arbor, USA; 6 Department of Electrical Engineering and Computer Science, University of Michigan, Ann Arbor, USA; 7 Michigan Center for Integrative Research in Critical Care (MCIRCC), University of Michigan, Ann Arbor, USA

**Keywords:** Ensemble learning, Deep learning, Medical image segmentation, X-ray coronary angiography

## Abstract

**Background:**

Automated segmentation of coronary arteries is a crucial step for computer-aided coronary artery disease (CAD) diagnosis and treatment planning. Correct delineation of the coronary artery is challenging in X-ray coronary angiography (XCA) due to the low signal-to-noise ratio and confounding background structures.

**Methods:**

A novel ensemble framework for coronary artery segmentation in XCA images is proposed, which utilizes deep learning and filter-based features to construct models using the gradient boosting decision tree (GBDT) and deep forest classifiers. The proposed method was trained and tested on 130 XCA images. For each pixel of interest in the XCA images, a 37-dimensional feature vector was constructed based on (1) the statistics of multi-scale filtering responses in the morphological, spatial, and frequency domains; and (2) the feature maps obtained from trained deep neural networks. The performance of these models was compared with those of common deep neural networks on metrics including precision, sensitivity, specificity, F1 score, AUROC (the area under the receiver operating characteristic curve), and IoU (intersection over union).

**Results:**

With hybrid under-sampling methods, the best performing GBDT model achieved a mean F1 score of 0.874, AUROC of 0.947, sensitivity of 0.902, and specificity of 0.992; while the best performing deep forest model obtained a mean F1 score of 0.867, AUROC of 0.95, sensitivity of 0.867, and specificity of 0.993. Compared with the evaluated deep neural networks, both models had better or comparable performance for all evaluated metrics with lower standard deviations over the test images.

**Conclusions:**

The proposed feature-based ensemble method outperformed common deep convolutional neural networks in most performance metrics while yielding more consistent results. Such a method can be used to facilitate the assessment of stenosis and improve the quality of care in patients with CAD.

## Background

As the most common type of heart disease, coronary artery disease (CAD) is the leading cause of death globally, resulting in a yearly loss of 17.9 million lives with 330 million being affected [1, 2]. CAD is primarily caused by the narrowing of the lumen in coronary arteries due to plaque build-up [3]. This narrowing, or stenosis, restricts the blood flow to cardiac muscle, depriving the heart of oxygen and nutrient supplements, ultimately leading to myocardial ischemia and infarction [4].

X-ray coronary angiography (XCA) is the gold standard for CAD diagnosis [5]. By releasing dye into the coronary vessels and inspecting its flow through the vessel structure via 2D projections, XCA helps clinicians locate potential stenoses, visually measure their severity, and determine the appropriate interventional therapies [6].

Visual stenosis assessment, however, is often unreliable: it tends to overestimate severe blockages while underestimate mild ones [7, 8] and has high intra- and inter-observer variability [9, 10]. To evaluate the lumen diameter more objectively, quantitative coronary angiography (QCA) was introduced [11] to offer a (semi-)automatic analysis of XCA. QCA analysis involves frame selection, vessel segmentation, stenosis positioning, and quantitative measurement [12, 13]. The vessel segmentation step of QCA is a prerequisite for calculating the percentage of arterial stenosis. Moreover, the correct delineation of coronary arteries plays an important role in center-line extraction, which is used for 3D reconstruction of blood vessels [14], vessel tracking [15], and cardiac dynamics assessment [16].

Due to the nature of XCA images, segmenting vessels accurately is challenging. First, XCA images usually are of low resolution, have low signal-to-noise ratios, and exhibit low contrast between the vessel structure and background region [17–19]. Second, the presence of irrelevant structures such as the catheter, diaphragm, and the spine is confounding and leads to non-uniform illumination within the images [20]. Third, the various angles from which the 3D vessel structure is projected to form 2D XCA images create twisted and overlapping vessels, making the segmentation even more challenging [21].

To overcome these difficulties and aid in the quantitative diagnosis of CAD, efforts have been made to develop both supervised and unsupervised methods for automatic coronary vessel segmentation.

Unsupervised methods can be primarily categorized as tracking-based, model-based, or filter-based [22]. Tracking-based methods [23] choose seed points on the edges and the center-lines of vessels, then take a small step in the direction of the vessel to look for the vessel edges or the center-lines nearby. When new edges are found, an estimate of vessel direction is made to take the next step in this search direction. Model-based methods [24–29], use deformable models or region growing to evolve the segmentation towards the vessel-background boundaries based on the forces and constraints defined by energy functions. They may also apply growing conditions defined by similarity functions together with a threshold parameter. Both tracking-based and model-based methods require initial seeds for segmentation and are therefore sensitive to initialization. Although they tend to maintain good segmentation continuity for the vessel tree structure, they may fail to handle confounding elements in the background that are adjacent to the vessels. Filter-based methods [17, 30] apply a variety of filters for non-uniform background intensity balancing, irrelevant structures suppression, noise reduction, and vessel enhancement. The filtered images can be later processed with thresholding techniques for segmentation mask generation. Due to their ease of implementation and their ability to mitigate illumination problems, filter-based methods have also been employed extensively as preprocessing steps in both supervised and unsupervised methods for automated coronary vessel segmentation [29, 31–36]. However, they are usually insufficient for use on their own, as they are sensitive to background structures and may not perform well on vessel junctions and bifurcations [37].

Coronary artery segmentation using supervised methods can be considered as a pixel-wise classification problem, with most current methods utilizing Neural Networks. Cervantes-Sanchez et al. [31] trained a multilayer perceptron with XCA images enhanced by Gaussian matched filters and Gabor filters. Nasr-Esfahani et al. [32] presented a multi-stage model where a Convolutional Neural Networks (CNNs) extracts local, contextual, and edge-based information that were then combined via a final fully connected layer. Recently, deep learning approaches have gained popularity in segmenting both major arteries and full artery trees from XCA images. Samuel and Veeramalai [38] proposed a Vessel Specific Skip Chain Network by adding two vessel-specific layers to the VGG-16 network [39]. Jo et al. [33] developed a two-stage CNN specifically for left anterior descending artery segmentation, where the first stage located candidate areas of interest and the second stage generated the segmentation mask. Iyer et al. [40] designed an angiographic processing network that learned how to preprocess the XCA images with the most suitable filters for local contrast enhancement. The preprocessed images were then fed into DeeplabV3+ [41] for segmentation. Shi et al. [42] developed a generative adversarial network for major branch segmentation with a U-Net generator and a pyramid-structure discriminator, reporting improved connectivity for the segmented mask. Yang et al. [43] replaced the backbone of U-Net with an ImageNet pre-trained ResNet [44], InceptionResNetv2 [45], or DenseNet [46] for main branch segmentation. Fan et al. [47] modified U-Net so that the proposed structure can receive both the target and registered background images before dye release as inputs for generating segmentation masks. The network structure proposed by [48] receives multi-channel inputs by adding a 3D convolution layer to the U-Net encoder, exploiting the temporal information using three consecutive frames from angiographic image sequences to produce a segmentation mask for the middle frame. Zhu et al. [49] applied the Pyramid Scene Parsing Network, a network proposed by [50], for coronary vessel segmentation. They took advantage of the network structure to incorporate features from multiple scales by pyramid pooling and used transfer learning to avoid overfitting on a small training set. Supervised methods for coronary artery segmentation may focus on the major coronary arteries for which clinicians would be more concerned, instead of the entire arterial tree. Network-based supervised methods have a number of drawbacks, including overfitting when the training set is small, weaker interpretability as compared to unsupervised filter-based methods, and an inability to ensure connectivity within their prediction masks. However, supervised methods require less manual input and are more robust in discriminating background structures such as the catheter and spine than unsupervised methods.

In this paper novel ensemble framework for coronary artery segmentation is proposed that employs gradient-boosting decision tree (GBDT) [51] and Deep Forest classifiers [52]. The GBDT is a popular machine learning technique that combines weak decision tree learners for loss function minimization. When constructing a GBDT model, a series of trees is built wherein each new weak decision tree attempts to correct errors from the previous stage. The Deep Forest classifier, on the other hand, is a deep ensemble model that uses non-differentiable modules to form deep learning structures. Unlike deep neural networks, it does not apply back-propagation for training, but it still uses multiple layers (with cascade structures) for processing and applies in-model feature transformation. However, GBDT boosts the performance of weak learners gradually in a sequential and additive way, while in Deep Forest, random forests composed of decision trees are considered as a subroutine stacked by layers, with layer outputs feeding into another layer to create depth. Though both GBDT and Deep Forest have not been applied to XCA image segmentation, they have been recently employed in medical image analysis in different image modalities [53–58]. The ensemble methods produced promising results in retinal vessel segmentation [59–64] and have not been, as far as we know, applied on coronary artery segmentation yet. In this study, 16 deep learning features obtained from the last layer of the Dense-Net-backbone U-Net decoder were combined with 21 multi-scale statistics on responses to a diverse range of filters to construct a 37-dimensional feature vector for each pixel in the input XCA image for training coronary artery segmentation models with GBDT and Deep Forest.

The proposed work takes advantage of both decades of classical computer vision research along with contemporary machine learning and deep learning techniques by employing a diverse set of reliable, well-established, hand-crafted features together with features from a deep structure for ensemble model training. Additional novelties come from the extraction of multiple statistics from the scale-space profile of a filter response and the adoption of a deep ensemble model on coronary artery segmentation.

The remainder of the paper is organized as follows. “Methods” section introduces the datasets used in this study and describes the methods employed for feature extraction, the under-sampling of imbalanced training classes, and model training, testing, and evaluation. “Results” section reports the effect of under-sampling on the training set, the performance of models constructed using different classifiers, and the analysis of feature importance, while “Discussion section provides interpretations of the results and describes limitations and future directions of the current work.

## Methods

**Fig. 1:**
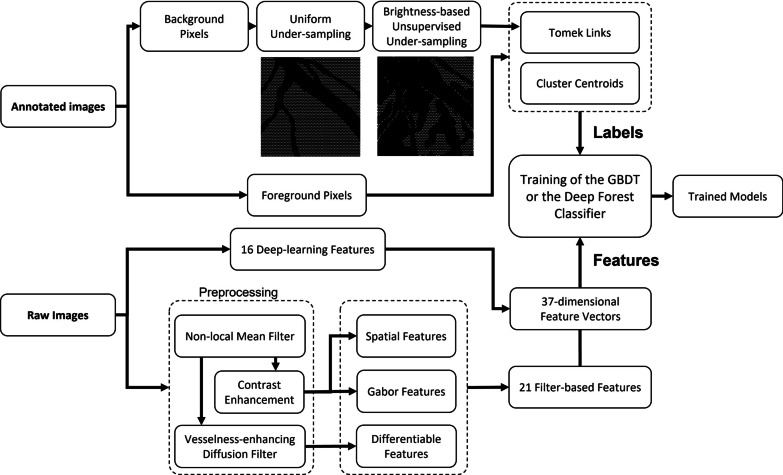
Schematic diagram of the training pipeline. Lower panel: features were extracted from raw images with deep-learning and filter-based methods. Upper panel: under-sampling methods were performed to balance the number of positive (vessel) and negative (background) training classes

In the following subsections, the datasets used for the study, the feature extraction techniques (using filter-based and deep learning methods) and under-sampling methods employed are first introduced, after which the training of ensemble classifiers is explained. A schematic diagram of the proposed method for coronary artery segmentation is depicted in Fig. 1.

### Dataset

The study was conducted with de-identified angiograms from two sources: “Dataset 1”, collected from the University of Michigan Hospital, Ann Arbor, MI and “Dataset 2”, collected from a hospital in the United Kingdom . Both datasets are comprised of patients suspected of having coronary artery disease who underwent invasive coronary angiography. Dataset 1 contains three subsets: 1-17, 1-19, and 1-AVI. Angiograms within subsets 1-17 and 1-19 were collected in 2017 and 2019, respectively, and stored in DICOM (Digital Imaging and Communications in Medicine) format, while angiograms in 1-AVI were stored in AVI (Audio Video Interleave) format.

Patients were excluded if any of the following occurred: incomplete injection of contrast dye, percutaneous coronary intervention, an implanted pacemaker or cardioverter defibrillator, or the presence of artificial objects other than the dye injection catheter. Ultimately, 130 angiogram sequences from 130 patients were included in this study. 80 of them visualize the left coronary artery (LCA) while the remaining 50 depict the right coronary artery (RCA). The number of frames in each sequence ranges from 43 to 150, with an average of 86 frames per sequence.

**Fig. 2:**
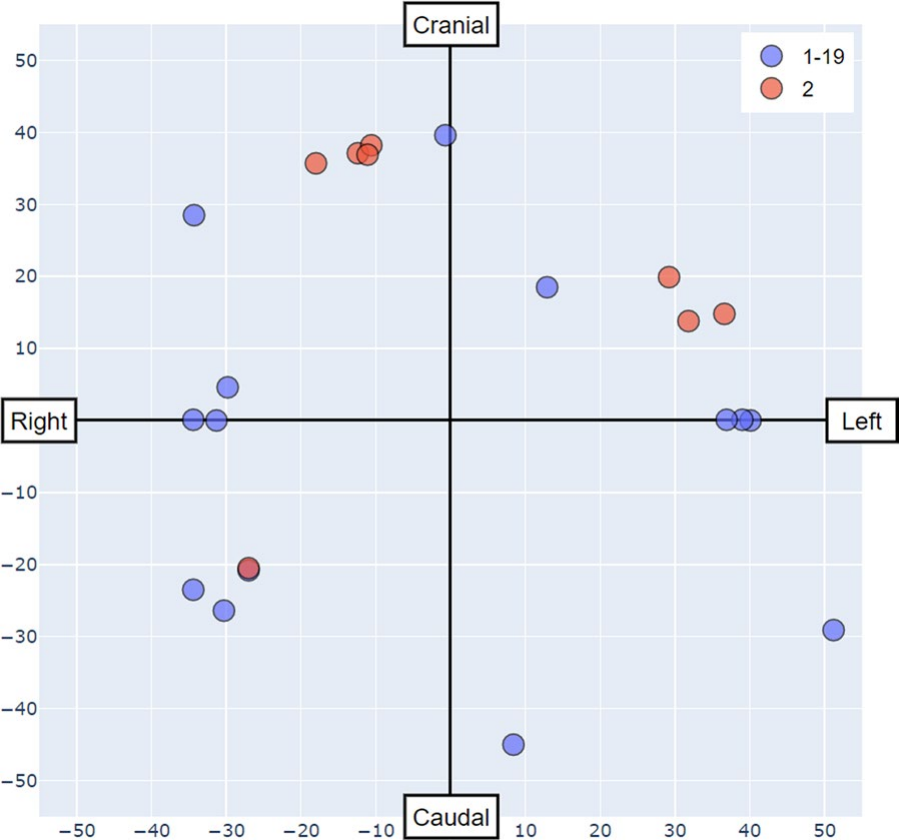
Image acquisition angles for Dataset 1-19 and Dataset 2. “Caudal” and “Cranial” refer to the caudal and cranial angulation of the X-ray

As the entire vascular tree is not always visible in all frames, frames were selected from XCA videos according to three criteria: (1) the selected frame contains the full injection of contrast agent; (2) there is minimal cardiac motion between adjacent frames; and (3) the full coronary artery is visualized in the frame. The frames are gray-scale images with a resolution of $512\times 512$ pixels. Segmentation masks used for training and testing were first generated manually using Adobe Photoshop CS and later validated by experienced cardiologists. Catheter diameter size (measured by pixel number and referred to as “Cath” in later sections) was recorded along with the annotation. Only those vessels whose diameters were greater than or equal to $0.75 \times$ Cath were annotated. The mean diameter of Cath was 8.0 (± 1.2) pixels. A summary of the dataset information is listed in Table 1. For angiograms whose acquisition angles are available, the information is depicted in Fig. 2.

**Table 1:** Dataset summary

Dataset code	Total	LCA	RCA	With acquisition angles
1-17	98	68	30	0
1-19	8	4	4	8
1-AVI	10	0	10	0
2	14	8	6	14
Total count	130	80	50	22

### Feature extraction with filters

#### Scale-space theory and the Z-profile of filter responses

**Fig. 3:**
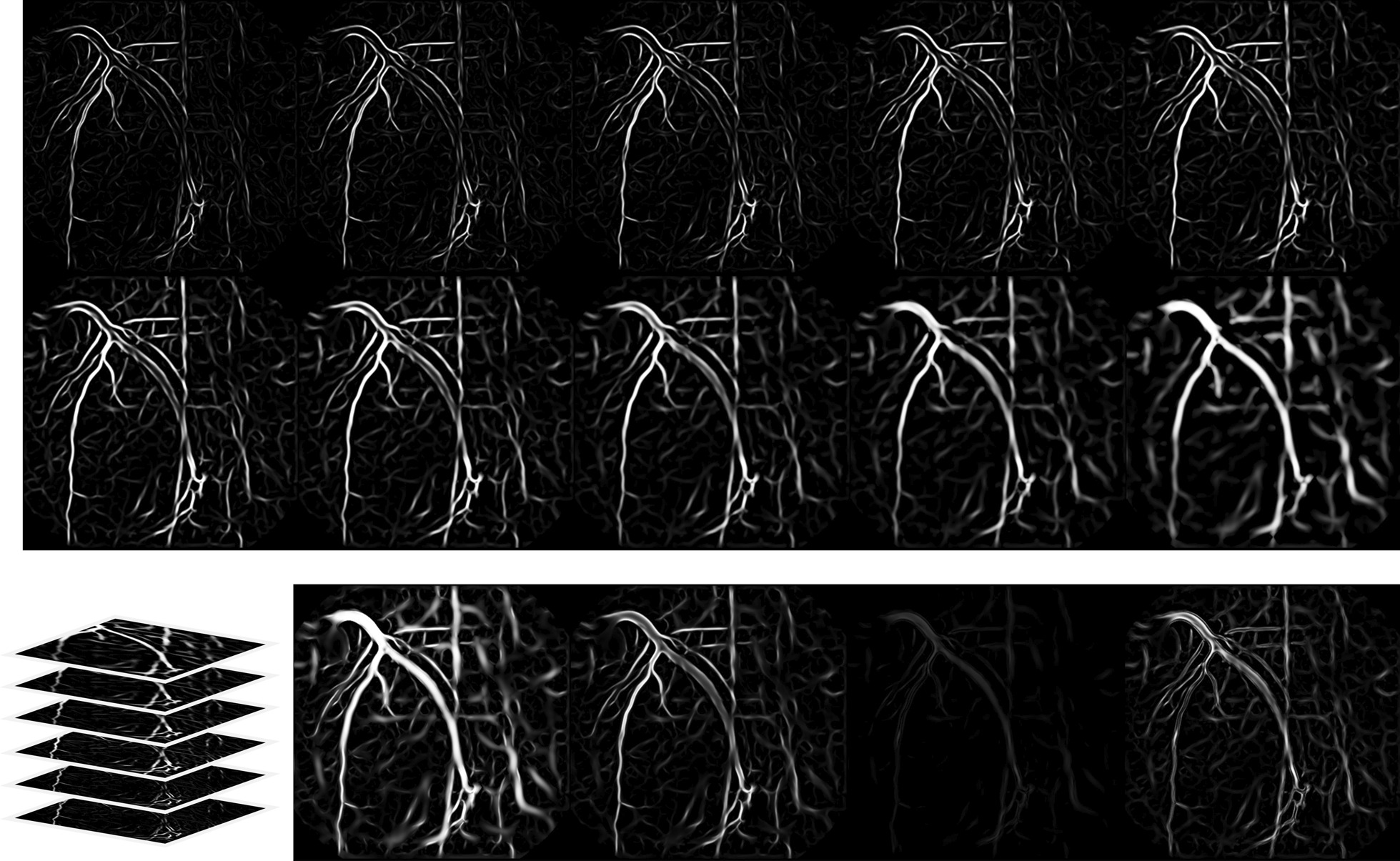
Multi-scale filtering with Frangi filter (upper panel) and the corresponding Z-profile of the max, mean, variance, and interquartile range of filtering responses (lower panel, from left to right)

Structures that a filter can extract from an XCA image depend on the scale of observation. A single scale is not always sufficient for capturing vessel structures of varying sizes. The scale-space theory [65] provides a framework for automatic scale selection in image filtering by applying multiple scales for image representation and summarizing filter responses across scales [66]. Based on this theory, the Z-profile of pixel-wise filter responses is constructed with four summary statistics: the maximum, mean, variance, and interquartile range of multi-scale responses. For example, Fig. 3 illustrates the filter response of Frangi filters [67] over ten different scales and the Z-profile thus obtained.

**Fig. 4:**
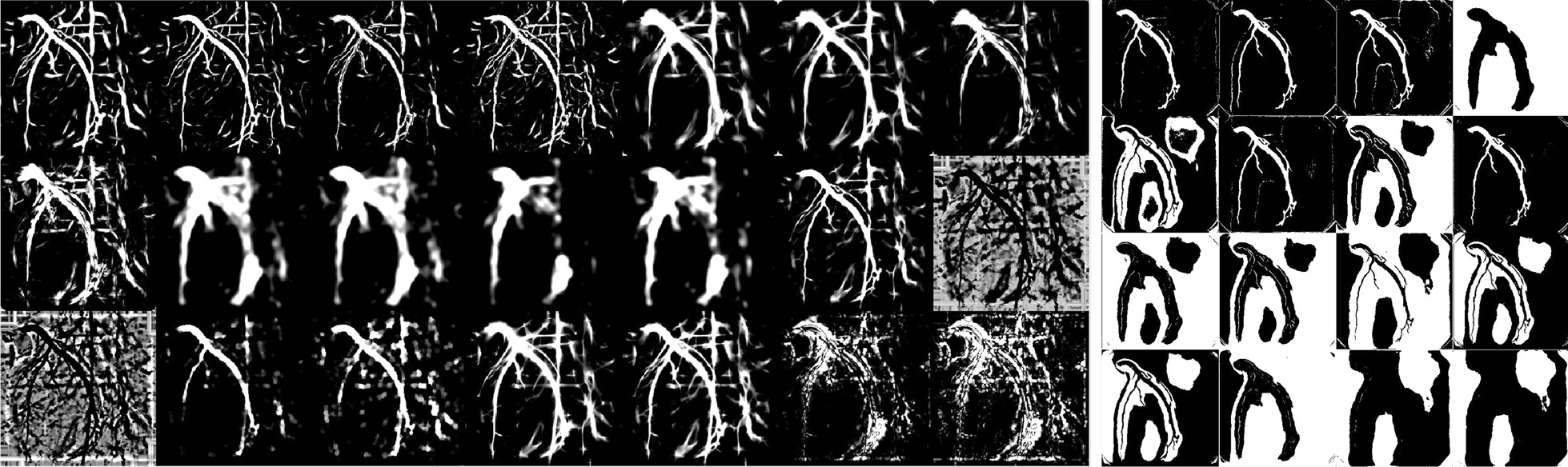
An example of the 37-dimensional feature maps extracted by filter-based methods (left panel; $3\times 7=21$ features) and deep learning method (right panel; $4\times 4=16$ features)

Scale ranges ($\lambda \in \Lambda$) were selected to be relative to the physical constraints of coronary arteries, ranging from 0.66$\times$Cath to 6.33$\times$Cath on a logarithmic scale.

#### Preprocessing

XCAs are often of poor quality due to image noise. To enhance the visibility of vessels within the frame, standard computer vision and image processing techniques were used to construct the preprocessing pipeline. First, metadata from the DICOM files was used to exclude the border regions (pixels outside of the imaging window) from analysis and to obtain catheter size information. Then, the filter scales were set as described in “Scale-space theory and the Z-profile of filter responses” section. In cases where the aforementioned metadata was unavailable, the mean Cath value (“Dataset” section) was used. After that, a non-local mean filter [68] was applied for noise reduction. Following this step, contrast adjustment (Algorithm 1) using Top-bottom-hat filtering [69] was employ to reconstruct the image.

Let $I:\Omega \rightarrow {\mathbb {R}}$ be the $H\times W$ image with pixel coordinates given by $(x,y)\in \Omega =\{1,2,\ldots ,H\}\times \{1,2,\ldots ,W\}$ and $SE_{\lambda }$ denote the structuring element (SE) with scale $\lambda$, then the Top-hat filtered image $I_{top}$ is defined as the maximum of the differences between an input image *I* and its SE opening over $\lambda \in \Lambda$, while the Bottom-hat output $I_{bottom}$ is defined as the maximum of the differences between SE closing with *I* over $\lambda \in \Lambda$, that is,

$$\begin{aligned} I_{top}&= \max _{\lambda \in \Lambda } \left( I - ( I \circ SE_{\lambda }) \right) \mathrm {and} \\ I_{bottom}&= \max _{\lambda \in \Lambda } \left( ( I \bullet SE_{\lambda }) - I\right) , \end{aligned}$$

with ($\circ$) and ($\bullet$) denoting morphological opening and closing respectively (see Appendix 6.1 for the definitions of these operations.) The Top-bottom-hat enhanced image is then generated as

1 $$\begin{aligned} I_{enhanced} = I + m\cdot I_{top} - n \cdot I_{bottom}, \end{aligned}$$

where *m* and *n* are the strengths of Top-hat and Bottom-hat transformations. 
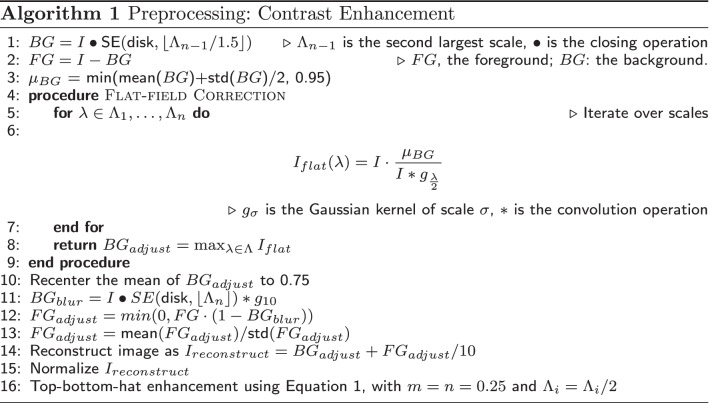


Vesselness-enhancing diffusion filtering [70] (see Appendix 1 for details) was also performed on the denoised images to enhance vascular structures as utilized in “Filter-based feature extraction” section.

#### Filter-based feature extraction

A number of common vessel enhancement and segmentation filters were used to extract features that can be categorized into differentiable, spatial, and Gabor features.

In terms of differentiable features, the Z-profile of the Frangi filter [67], Z-profile of the matched filter [71], the Gaussian-filter-smoothed Z-profile of the gradient magnitude, and the vessel confidence measure [72] were extracted, resulting in a total of 13 features. For spatial features, the granular decomposition of the top-bottom-hat image using the method given in [73] was obtained, producing a Z-profile with 4 features. The Gabor features were extracted from the Z-profile of the Gabor filter [17] responses on the complement of contrast enhancement output image.

### Feature extraction with deep learning

The deep learning networks described in this section were implemented in Python 3.7 using PyTorch 1.10 and the segmentation model package [74]. Each network was trained on a single NVIDIA Tesla V100 GPU.

#### Data partitioning for model construction

The dataset containing 130 XCA images was split into training, validation, and test sets in 3:1:1 ratio. The partitions were stratified to ensure different subsets had approximately the same percentage of samples of RCA and LCA angiograms from different sources.

#### Network structure

The network structures adopted in this study are common deep learning models developed for medical image segmentation, such as U-Net, DeepLabV3+, InceptionResNet-v2-backbone U-Net, ResNet101-backbone U-Net, and DenseNet121-backbone U-Net. The latter three were first applied for main branch segmentation in XCA images in [43] and achieved the best performance in terms of F1 score thus far. These structures were employed in this work for major branch segmentation with modified training logic to serve as comparison to the ensemble models, and to obtain deep learning features for ensemble model training. For the DeepLabV3+ model, we used a DeepLabV3 encoder as mentioned in [41] and adapted the ImageNet pre-trained ResNet101 [44] for dense features extraction in the encoder [75]. Details on model parameters can be found in Appendix 3. For the modified U-Net models, the encoder and bottleneck sections of the U-Net were replaced with ImageNet pre-trained ResNet [44], InceptionResNet-v2 [45], or DenseNet [46], respectively, except for their average pooling layers and the fully connecting layers at the end. Skip connections were retained between the encoder and the decoder at different spatial resolutions.

#### Data processing and training setting

Gray-scale XCA images were first preprocessed with 2-D min/max normalization. To increase the diversity of the training samples, data augmentation was employed at each training iteration before feeding data into the networks. Specifically, XCA images were randomly augmented by affine transformations ($-20^\circ$ to 20$^\circ$ rotation, 0–10% of image size translation shift on horizontal and vertical axes, or 0–10% zoom) with a probability of 0.7. The same augmentations were also applied to the corresponding ground-truth masks. The network was trained using a default-setting Adam optimizer with an initial learning rate of $10^{-3}$ and a mini-batch size of 8 images for up to 100 epochs. An early-stop mechanism was triggered if validation loss did not improve for 15 epochs.

#### Loss function

To take into consideration class imbalance and class importance, the deep-learning model was trained using Generalized Dice loss (GD) [76]

$$\begin{aligned} GD = 1 - \frac{2 \sum _{c=1}^t w_c \sum _{p=1}^n G_{cp} M_{cp}}{\sum _{c=1}^t w_c \sum _{p=1}^n (G_{cp}+M_{cp})}, \end{aligned}$$

where $w_c = \left(\sum ^n_{p=1}(G_{cp}/t)) ^2 + \epsilon \right)^{-1}$ is the weight for class *c*, *t* is the total class number, *p* is the pixel location, and *n* the total number of pixels in an image. $G_{cp}$ and $M_{cp}$ are the ground truth image pixel and predicted mask pixel values from class *c* respectively.

#### Testing and model evaluation

For each network structure, the model that achieved the lowest validation loss was applied to the test set. The final layer of the network output was passed through an element-wise sigmoid activation function to generate the probability of each pixel belonging to either the vessel region or the background region. The same post-processing described in “Post-processing and model evaluation” section was applied to generate the final binary segmentation masks. The quality of the generated masks was evaluated by precision, sensitivity, specificity, F1 score, Intersection over Union (IoU), and the Area Under the Receiver Operating Characteristic Curve (AUROC). The first five are defined as

$$\begin{aligned} Precision&= \frac{TP}{TP+FP} \\ Sensitivity\,(Recall)&= \frac{TP}{TP+FN} \\ Specificity&= \frac{TN}{TN+FP} \\ F1\;Score&= \frac{2\times Sensitivity \times Precision}{Sensitivity + Precision} \\ IoU&= \frac{TP}{TP + FP + FN}, \end{aligned}$$

where TP, TN, FP, and FN are the pixel counts of true positives, true negative, false positives, and false negatives, respectively.

#### Deep feature extraction

The network structure that achieved the best test performance with respect to F1 score and AUROC was adopted for deep feature extraction. After normalization, the XCA images of dimension $1\times 512\times 512$ were fed into the network and the activation maps of the final decoder layer were extracted as the deep features with dimension of $16\times 512\times 512$.

### Feature standardization and training samples

After feature extraction as described in Feature extraction with filters and Feature extraction with deep learning” sections, all features (Table 2) were concatenated (Fig. 4). Each feature map outputted from a filter or a deep learning layer is standardized individually by subtracting its mean pixel value and dividing the result by the standard deviation. For each pixel $p_i$ within an XCA image, a 37-dimensional feature vector $\varvec{X_i}$ was extracted that yielded a training sample ($\varvec{X_i}$, $y_i$) where the label $y_i$ is 1 if the pixel is part of a vessel in the annotated XCA image and 0 otherwise. The 130 XCA images resulted a in total of $512\times 512 \times 130 = 34078720$ samples before border removal and any under-sampling.

**Table 2:** Feature domains and types

Feature domain	Feature type	Feature number
Differentiable features	Z-profile of Frangi filters	4
	Z-profile of matched filters	4
	Gaussian-filter-smoothed Z-profile of the gradient magnitude	4
	Vessel confidence measure	1
Spatial features	Z-profile of granular decomposition	4
Gabor features	Z-profile of Gabor features	4
Deep-learning features	Activation maps of the final decoder layer	16

### Under-sampling of non-vessel pixels

If vessel and background pixels are denoted as positive and negative classes respectively, the samples are highly imbalanced as the minority (positive) class only comprises an average of $5.58(\pm 1.99)\%$ in the XCA images. Moreover, the features extracted between neighboring pixels are highly correlated. Given these two facts, hybrid under-sampling of pixels at the image level was performed to (1) avoid over-fitting due to redundant information across pixels; (2) ensure that the classifiers do not ignore the minority class; and (3) reduce training time.

#### Uniform and unsupervised under-sampling

**Fig. 5:**
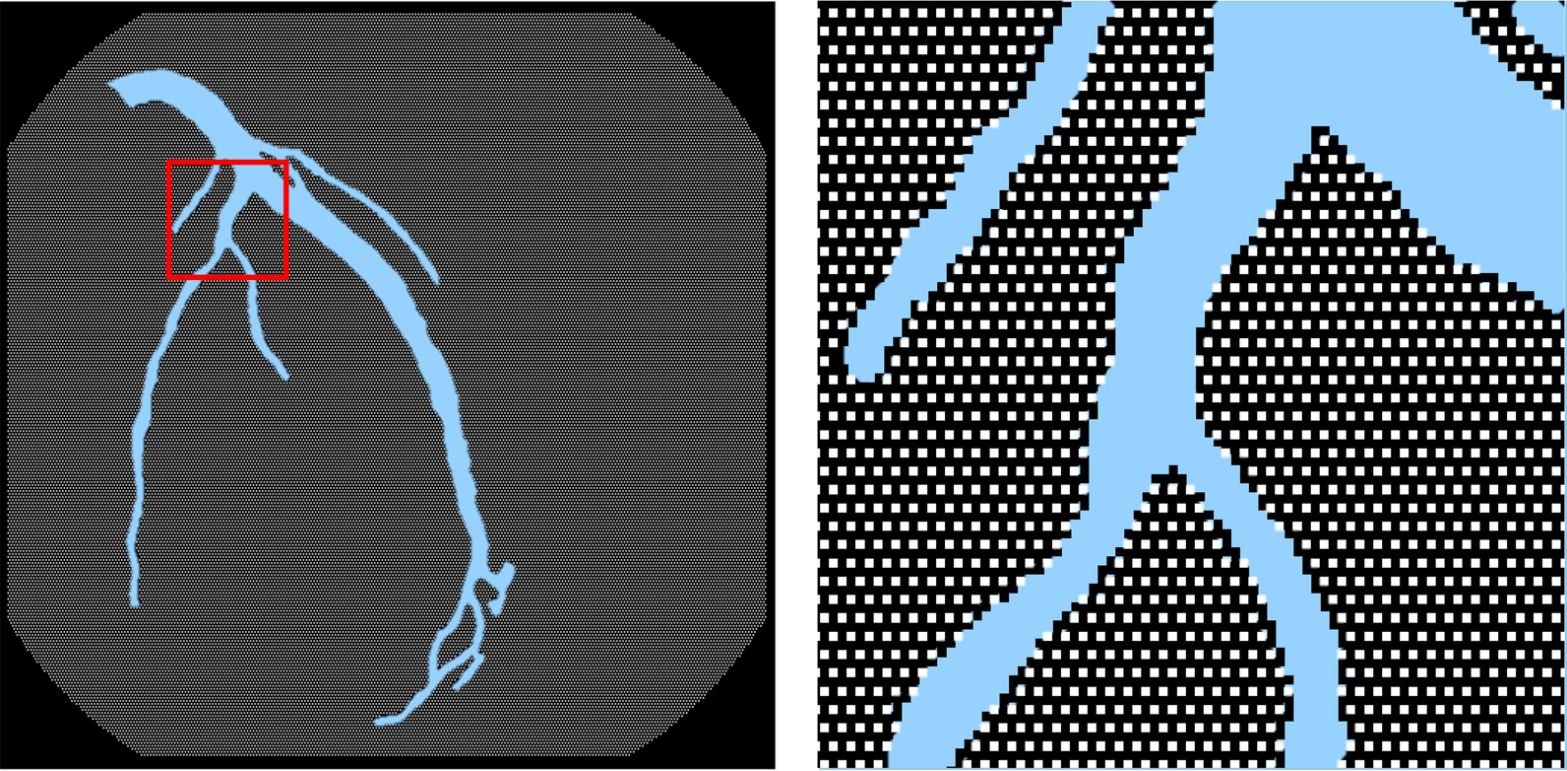
A uniform under-sampling mask of the majority class. Pixels from the minority class colored light blue are not involved. The mask image on the right is a magnified version of the selected red box on the left. Pixels colored white were retained after the mask was applied to major class of the target image

**Fig. 6:**
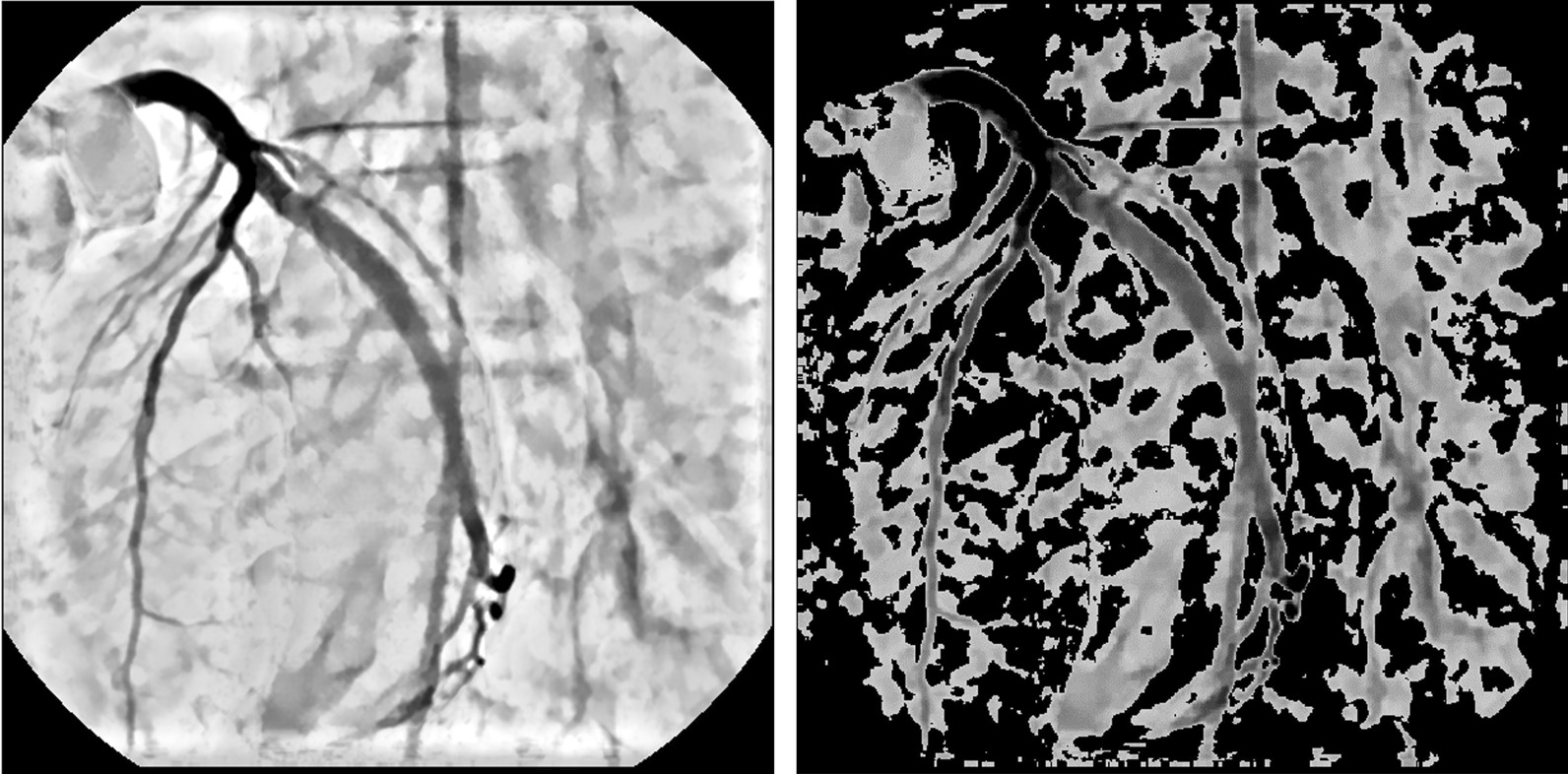
Unsupervised under-sampling. Left: the output image from contrast enhancement; Right: Pixels retained after under-sampling based on intensity

The majority class consists of the background, non-vessel areas in the XCA images. Pixels from the majority class were first uniformly under-sampled via a mask (Fig. 5) such that the 8-neighborhood of each sampled pixel was not sampled. Then, an intensity-based unsupervised under-sampling method was employed to further reduce the major class based on the contrast enhancement output (Fig. 6). To affect this under-sampling, the histogram of the pixel intensity of the contrast enhanced image was created with 256 bins, after which the discrete pdf (probability density function) was obtained and assigned to a one-dimensional median filter. The smoothed pdf was compared with the original one to identify the over-saturation peak generated by contrast enhancement. When these over-saturated pixels were excluded, the median value of pixel intensities was calculated as the binarization threshold for bright pixel removal (Fig. 6, right panel). This step remove pixels that clearly belong to the background based solely on their intensity.

#### Supervised under-sampling

**Fig. 7:**
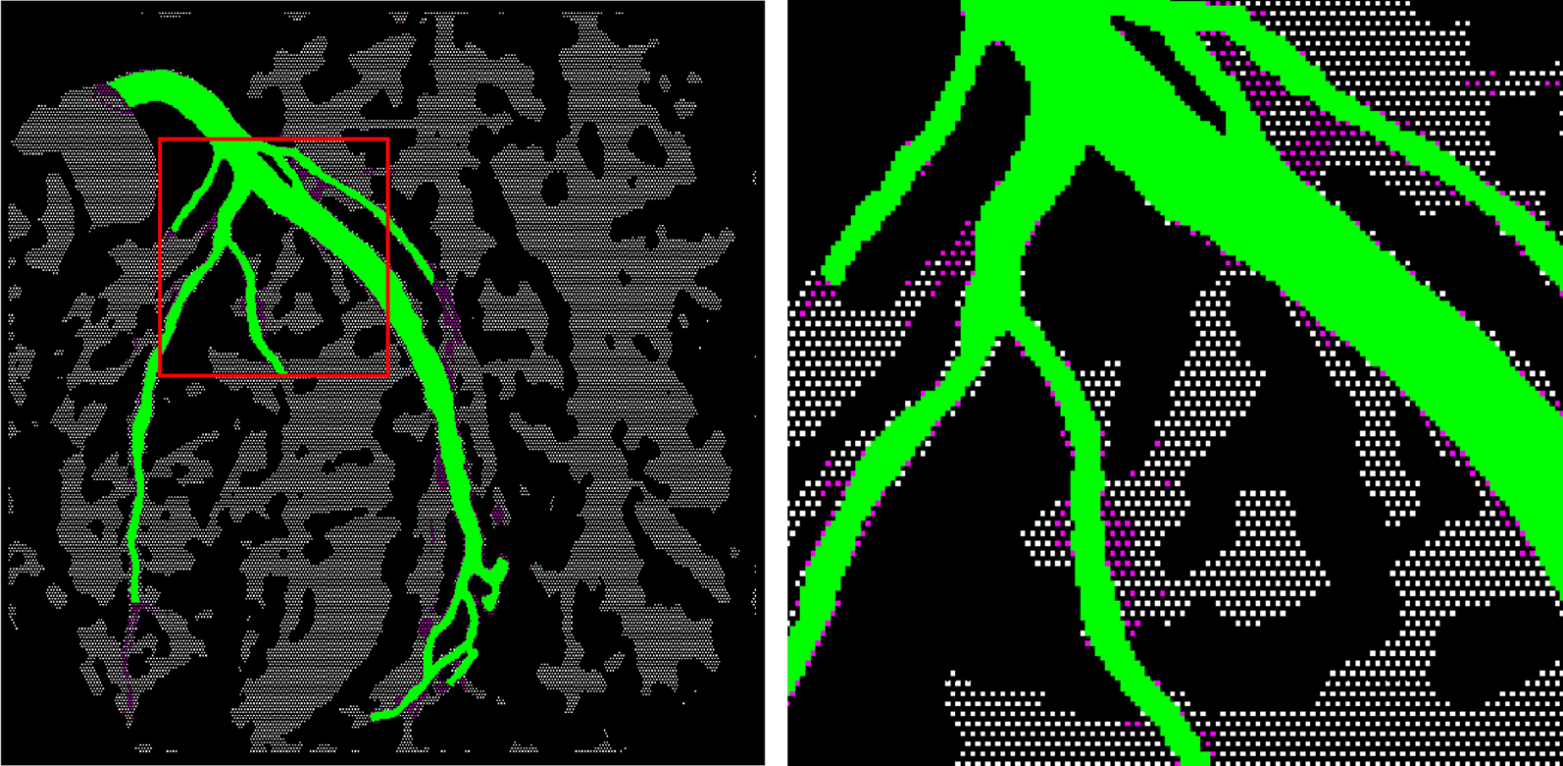
Tomek Links under-sampling. The image on the right is a magnified version of the red box on the left. Magenta: pixels removed by Tomek Link; Green: positive class, vessel pixels; White: negative class, background pixels

Supervised under-sampling includes Tomek Links and Cluster Centroid under-sampling for both the positive and the negative classes. Tomek Links [77] are defined as pairs of pixels from opposite classes that are the nearest neighbors of each other. As removing overlapping pixels between classes yields more well-defined boundaries for the classifiers [78], the Tomek Links of both classes were removed so that the minimally distanced nearest-neighbor pairs of vessel pixels belong to the same class. Figure 7 illustrates an Tomek Link under-sampling. Following the Tomek Links, the Cluster Centroid [79] under-sampling method was employed. This method first applies clustering algorithms such as *k*-nearest neighbors to generate cluster centroids and then uses these centroids to replace the original sample points. This method can reduce the number of pixels within each class to a fixed number, e.g., 4000, which is much smaller than the sample number in the original image. The Python implementation [80] of Tomek Links and Cluster Centroid methods were employed.

### Ensemble learning for coronary vessel segmentation

Two ensemble methods, Gradient Boosting Decision Tree (GBDT) [51] and Deep Forest [52] were trained on the extracted 37-dimensional feature vectors with samples obtained by under-sampling. To explore how Tomek Links and Cluster Centroid affected the segmentation performance, models with and without these two under-sampling methods were constructed for comparison.

#### Data partitioning for model construction

The dataset was split into training and test sets with a 4:1 ratio. The test set images are exactly the same as the deep learning test set mentioned in “Data partitioning for model construction” section and were not utilized until the test stage. All the partitions (including the cross-validation partitions in “Training and testing strategy” section) were stratified to ensure different sets have approximately the same percentage of LCA and RCA images, and pixels from the same image were not split across sets. Moreover, standardization was applied to the training set with parameters being saved to transform the test set, and this was also true for cross-validation.

#### Training and testing strategy

For the Deep Forest model, default hyper-parameters settings were used since hyper-parameters had minimal effect on the model performance [52], while for the GBDT model, hyper-parameters were tuned using 4-fold cross-validation, where fold compositions were changed under cyclic permutation with a 3:1 ratio. These hyper-parameters and their respective ranges included the learning rate ($\{0.01, 0.05, 0.1\}$), the number of boosting stages ($\{100, 500, 1000, 2000\}$), and the maximum depth of the individual regression estimators ($\{3, 5, 10, 20\}$). For other hyper-parameters, default value in the Sklearn package were applied. For example, the loss function to be optimized was the deviance and the split quality of trees were measured by the mean squared error with improvement score by Friedman (Friedman MSE) [51]. Finally, the hyper-parameter combination that achieved the highest mean AUROC score in cross-validation was used to train the final GBDT model on the training set and applied to the test set for evaluation.

#### Post-processing and model evaluation

Features extracted from test images were passed into the trained ensemble models to generate masks. The masks were then binarized using Otsu’s method [81] and post-processed by (1) removing border regions, (2) adding back unsupervised background masks, and (3) removing artifacts whose areas values were less than 50. The same evaluation metrics listed in “ Testing and model evaluation” section were calculated to evaluate the quality of the predicted masks across different models. The metrics were calculated image-wise and produced by calculating the mean and standard deviation over all tested images.

#### Feature importance

The permutation importance [82] of the GBDT models were computed on the holdout test set for feature evaluation. The importance of a feature was calculated as the decrease of Friedman MSE (mentioned in “Training and testing strategy” section) evaluated on the test set when permuting the feature column for 10 times.

## Results

### Under-sampling

The counts of positive and negative pixels retained for model training after different under-sampling steps are listed in Table 3. Before under-sampling, the positive class comprised only $5.56\%$ of the total samples after the border regions were removed (see “Preprocessing” section). The uniform under-sampling selected $24.13\%$ of the background pixels in the original labeled image, followed by the unsupervised under-sampling that further kept $45.98\%$ of the negative pixel samples. These two steps resulted in a $34.78\%$ share of vessel pixels in the overall samples and 4,000,602 samples of vessel and background pixels for model training. Tomek Links only altered this percentage level slightly, while Cluster Centroid completely balanced the positive and negative classes, reducing the training samples to 832,000 in total.

**Table 3:** Pixel totals resulting from different under-sampling methods

Pixel count after the method	% of positive class	% of negative class (the majority class)	Total count (training samples)
Original image (exclude border)	5.559	94.441	25,002,241
Uniform Under-Sampling	19.611	80.489	7,087,635
Unsupervised Under-sampling	34.663	65.338	4,010,003
Tomek links	34.677	65.323	4,000,602
Cluster centroid	50.000	50.000	832,000

### Performance comparison of deep-learning models and ensemble models on the test set

Table 4 lists the performances of five deep learning models, six ensemble models and the state-of-the-art deep learning model on the test set in terms of their precision, sensitivity, specificity, F1 score, AUROC and IoU. For the ensemble models, “Unsupervised” indicates that the uniform and unsupervised under-samplings were applied on the training samples while “Tomek Links” means that all the Tomek Links were also removed from the sample pixels. Moreover, “Cluster Centroid” indicates that the Cluster Centroid under-sampling method was further applied for reducing sample numbers.

**Table 4:** A comparison of model performance

	Precision	Sensitivity	Specificity	F1 Score	AUROC	IoU
U-Net	0.867 ± 0.073	0.810 ± 0.122	0.993 ± 0.005	0.831 ± 0.082	0.902 ± 0.060	0.719 ± 0.115
DeepLabV3+	0.862 ± 0.082	0.828 ± 0.096	0.992 ± 0.006	0.838 ± 0.081	0.909 ± 0.047	0.726 ± 0.088
Inception- ResNet-v2 U-Net	**0.904** ± **0.072**	0.805 ± 0.133	**0.995** ± **0.004**	0.842 ± 0.089	0.900 ± 0.066	0.737 ± 0.120
DenseNet121 U-Net	0.891 ± 0.053	0.824 ± 0.145	0.994 ± 0.004	0.845 ± 0.091	0.909 ± 0.071	0.741 ± 0.117
Resnet101 U-Net	0.865 ± 0.072	0.819 ± 0.122	0.992 ± 0.005	0.832 ± 0.068	0.906 ± 0.060	0.718 ± 0.095
Unsupervised with Deep forest	0.832 ± 0.073	**0.911** ± **0.096**	0.990 ± 0.005	0.863 ± 0.048	**0.95** ± **0.046**	0.762 ± 0.071
Tomek Links with Deep Forest	0.884 ± 0.061	0.867 ± 0.124	0.993 ± 0.004	0.867 ± 0.066	0.930 ± 0.061	0.770 ± 0.094
Cluster centroid with Deep forest	0.868 ± 0.067	0.873 ± 0.107	0.993 ± 0.004	0.864 ± 0.062	0.933 ± 0.053	0.765 ± 0.087
Unsupervised with GBDT	0.864 ± 0.066	0.894 ± 0.104	0.992 ± 0.004	0.872 ± 0.051	0.943 ± 0.051	0.776 ± 0.075
Tomek Links with GBDT	0.885 ± 0.06	0.872 ± 0.123	0.994 ± 0.004	0.870 ± 0.066	0.933 ± 0.060	0.775 ± 0.094
Cluster Centroid with GBDT	0.857 ± 0.073	0.902 ± 0.084	0.992 ± 0.004	**0.874** ± **0.048**	0.947 ± 0.041	**0.779** ± **0.072**
DenseNet121 U-Net [43]	0.858 ± 0.071	0.873 ± 0.109	0.991 ± 0.006	0.858 ± 0.057	0.926 ± 0.068	0.755 ± 0.082

Bold values are denotes the best-performing statistic of a metric among all models tested.

For the deep learning models, Inception-ResNet-v2 U-Net achieved the highest precision and specificity, DeepLabV3+ obtained the best sensitivity and AUROC, while DenseNet121 U-Net had the best F1 score, AUROC, and IoU scores. For ensemble learning models that use Deep Forest as the classifier, the samples after Tomek Links under-sampling method yielded the highest score in precision, specificity, F1 score, and IoU, while the samples after unsupervised under-sampling gave the best test performance in terms of sensitivity and AUROC, achieving the highest AUROC of 0.95 among all models tested. For ensemble learning models that use GBDT as classifier, the samples after Tomek Links under-sampling had the best precision and specificity. Moreover, the further application of the Cluster Centriod under-sampling method generated the highest sensitivity, F1 score, AUROC, and IoU scores. It also yielded the best F1 score and IoU of all models tested.

The state-of-the-art method [43] achieved higher sensitivity, F1 score, AUROC and IoU than the five deep learning models. However, it can not beat the proposed ensemble models, which generally performed better than the deep learning methods in all metrics except for specificity. Moreover, GBDT classifiers performed better than the Deep Forest with higher mean value and lower standard deviation in terms of F1 score and IoU regardless of the under-sampling method employed.

### The permutation feature importance of GBDT models

**Fig. 8:**
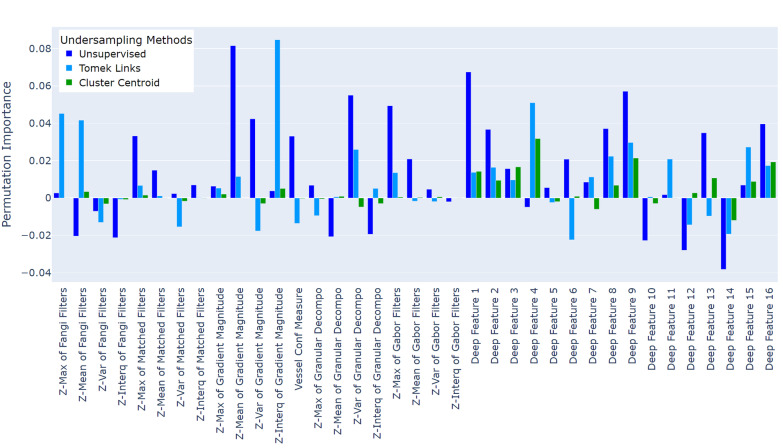
Permutation feature importance of GBDT models that were trained with different under-sampling methods. The smaller the value, the lower the importance

Figure 8 illustrates the permutation importance of the 37 features used in GBDT model training when different under-sampling methods were applied. Statistics (maximum, mean, variance, and interquartile range) of the Z-profile are denoted as Z-max, Z-mean, Z-var, and Z-interq in the plot. For the GBDT trained with the unsupervised under-sampling method, Z-mean and Z-var of the gradient magnitude, Z-var of the granular decomposition, and Z-max of the Gabor filter are importance filter-based features, as well as the deep features 1, 9, and 16. For the GBDT trained with Tomek Links under-sampling method, the Z-max and Z-mean of Frangi filter, the Z-interq of gradient magnitude, and deep feature 4 have high permutation importance. In terms of the the GBDT and Cluster Centriod combination, deep features 4, 9, and 15 show more permutation importance over other features. Filter-based features contribute less than the deep learning features when evaluated on the test set. Overall, GBDT model trained with different under-sampling methods assigned different contributions to features during test evaluation, with some features such as the Z-var and Z-interq of Gabor features, Z-var of Matched filters, Z-var and Z-interq of Frangi filters, deep features 5, 10, 12 and 14, having minor influence when evaluated by permutation importance.

## Discussion

In this paper, a novel ensemble framework for automatic segmentation of coronary arteries in XCA was developed. The best performing model utilized a GDBT classifier trained on samples generated by the Cluster Centroid under-sampling method, achieving a mean precision of 0.857, sensitivity of 0.902, specificity of 0.992, F1 score of 0.874, AUROC of 0.947, and IoU of 0.779. The ensemble methods outperformed DeepLabV3+, various U-Net-based models and the state-of-the-art method [43] on coronary vessel segmentation in almost all metrics and had a lower standard deviation in performance over test images.

From a clinical perspective, as more than 80% of the percutaneous coronary intervention (PCI) are performed at the time of angiography [83], an accurate vessel segmentation method that improves the quality of QCA can greatly facilitate the assessment of stenosis; improve the quality of patient care; and avoid unnecessary PCIs, yield billions of dollars in savings at the national level [84]. In addition, correct delineation of the coronary vascular structures would be valuable in many types of CAD such as coronary endothelial dysfunction, where XCA serves as a testing technique [85].

From a technical perspective, this is the first time to our knowledge that ensemble methods, especially deep ensemble methods, have been applied to coronary artery segmentation. Ensemble methods are known for reducing the variance of predictions by gathering weak learners. In this study, we specifically used them for better predictive performance and robustness with limited data. In terms of the ensemble methods applied, GBDT is one of the leading boosting algorithms that employ decision tree weak learners. Compared with other decision tree boosting algorithms (e.g. [86]), it has more flexibility to handle various losses defined in different forms. Deep Forest, on the other hand, is an emerging ensemble method that creates stacked layers in ensemble training. Although the Deep Forest classifier did not achieve performance that was significantly better than GBDT, it still outperformed the deep learning models and was more consistent when evaluated over all test images. This suggests that the ensemble learning method and the training framework in which various features were extracted from both classic and deep learning filters is more suitable for a relatively small dataset where training samples are limited. The significant increase in sensitivity indicates that the ensemble models have better recognition of the vessel area. This could be attributed to the employment of domain knowledge and a reduction in overfitting via the pixel-wise training scheme.

The proposed method deliberately introduces some redundancies within the feature vectors so that a wider spectrum of possible situations can be handled. In terms of future work, it is important to analyze the most discriminating features extracted for not only a more explainable model but also a more representative feature set. A review of the permutation importance of features indicates that some of the features have little contribution to the final prediction. However, since the permutation importance can be biased towards features that are correlated with one another [87], a more substantial feature analysis is required.

Different from the state-of-the-art paper on vessel segmentation [43], in which the dataset was run for 400 epochs with decreased learning rate on training loss saturation, we used an updated training logic to train the deep neural network model for feature extraction. Specifically, we reduced the training epoch upper bounds and introduced an early-stop mechanism. Although the deep models trained as such have inferior performances compared to those obtained from [43], we believe it is not necessary to have prolonged training for the following two reasons. First, with the training logic currently applied, the state-of-the-art method by itself did not outperform our ensemble model in all metrics. Second, the trained deep neural networks are only used for feature extraction. Given that our dataset is relatively small, prolonged training on the deep feature extraction model may hinder the generalizability of the ensemble model built on its top. To summarize, the ensemble methods we proposed have strengths in predictive power compared to the deep-learning state-of-the-art on our datasets. Comparatively, our weaknesses lie in the complexity of preprocessing, feature extraction, and under-sampling pipeline prior to model training.

Recently, vision transformer networks [88] have been introduced to tackle segmentation tasks [89] for their capability to capture long-range dependencies in images with the self-attention mechanism. However, most of the transformer-based networks are unable to be trained properly with a small-scale dataset. Current transformer-based structures designed for medical image segmentation either need thousands of annotated images [90] for training or require a large amount of computational resources [91]. Considering that we have limited training samples and a model that require a lot of computational resources is less operational at the point of care in the cardiac catheterization lab, the transform-based network may not be a practical or optimal choice.

The proposed method has several limitations. First, the current Python implementation of Cluster Centroid under-sampling requires much more computational time than the Tomek Links and the unsupervised under-sampling methods (see Appendix 2 for details). Although a huge reduction in training samples should expedite the training process and yield better-performing prediction models given the relatively small size of the datasets utilized in this study 4, the prolonged under-sampling process increases training time and may hinder the method’s efficiency and scalability in practice. Moreover, the choice of output pixel number for each class in the Cluster Centroid under-sampling method was determined through a number of trial experiments instead of a more comprehensive cross-validated grid search. Since this number affects the time required in under-sampling and the final performance, it is possible that better choices exist to reduce training time while maintaining a good performance.

## Figures and Tables

**Fig. 9:**
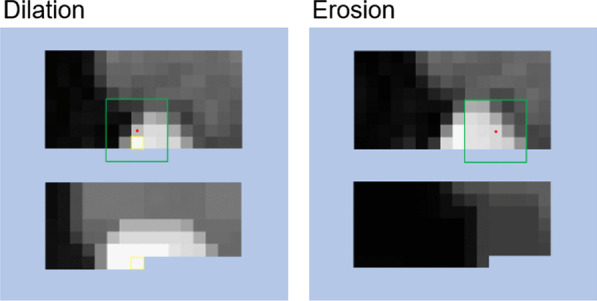
Examples of dilation (left) and erosion (right) on a grayscale image using a $5\times 5$ flat *SE* [92, 93]. The top and bottom parts illustrate the position and results of the structuring element window when applied on specific pixels of the original images

## Data Availability

Dataset 1 utilized in this study was collected at Michigan Medicine. The University of Michigan’s Innovation Partnerships unit (innovationpartnerships@umich.edu) will handle potential charges / arrangements of the use of data by external entities, using such methods as material transfer agreements.

## Appendix 1: Pre-processing

### Top-bottom-hat filtering

Top-hat and bottom-hat filters are morphological filters that combine dilation and erosion operations with a structuring element (SE). For a gray-scale image, dilation and erosion operation (Fig. 9) of a pixel return the minimum and the maximum, respectively, of the pixel intensities in its neighborhood defined by SE and hence, the former is often used for gaps filling and region connections, while the latter is applied for detail elimination. Denoting image matrix as *I* and the SE with scale $\lambda$ as $SE_{\lambda }$, the morphological opening operation ($\circ$) is define as an erosion ($\ominus$) followed by a dilation ($\oplus$) operation and the morphological closing operation ($\bullet$) first perform dilation and then erosion.

$$\begin{aligned} I \circ SE_{\lambda }&= (I \ominus SE_{\lambda }) \oplus SE_{\lambda } \\ I \bullet SE_{\lambda }&= (I \oplus SE_{\lambda }) \ominus SE_{\lambda } \end{aligned}$$

### Diffusion filtering

Diffusion is a time-dependent process from physics that models the concentration change. This process evolves the input frame, *I*, by introducing a ’time’ variable and generating images, *I*(*t*), evolved via the diffusion equation:

$$\begin{aligned} I(t) = \nabla \cdot (D \nabla I) \end{aligned}$$

with respect to time t. Here, $\nabla$ represents the divergence operator; $\nabla I = (I_x, I_y)$ denotes the image gradient; and *D* is a diffusion tensor that describes the diffusion process. The diffusion tensor is constructed to enhance the vascular structures in the image using a variant of Frangi’s vesselness filter with continuous derivatives. The filter $V_{\sigma }$ is given by

$$\begin{aligned} V_{\sigma } = {\left\{ \begin{array}{ll} 0, &{} \lambda _2 < 0 \\ exp \left( - \frac{R^2}{2r^2} \right) \times \left( 1 - exp \left(\frac{S^2}{2s^2}\right) \right) \times \left( - \frac{2c^2}{\lambda _2^2} \right) , &{} \lambda _2 \ge 0 \end{array}\right. } \end{aligned}$$

where $\lambda _1$ and $\lambda _2$ are the eigenvectors of the Hessian matrix, $|\lambda _2| > |\lambda _1|$, $R = \lambda _1 / \lambda _2$ and $S = \sqrt{\lambda _1^2+\lambda _2^2}$, the parameter $\sigma$ denotes convolution with a Gaussian Kernel of radius $\sigma$. The smoothing parameter, *c*, ensures that the derivative of $V_{\sigma }$ remains continuous at all points, ensuring a smooth function. The diffusion tensor must be smooth, positive definite, and symmetric. Due to the addition of the smoothing parameter in the above filter, the diffusion tensor can be defined as

$$\begin{aligned} D = Q G Q^T \end{aligned}$$

where Q is the matrix given by the eigenvectors of the Hessian of the image and

$$\begin{aligned} G = \begin{bmatrix} \lambda _1^{'} &{}\quad 0 \\ 0 &{}\quad \lambda _2^{'} \end{bmatrix} = \begin{bmatrix} 1 + (\omega -1)\cdot V^{s^{-1}} &{}\quad 0 \\ 0 &{}\quad 1 + (\epsilon -1)\cdot V^{s^{-1}} \end{bmatrix} \end{aligned}$$

In this definition, $\omega$, $\epsilon$, and *s* denote additional tuning parameters.

Vessel-enhancing diffusion is performed on each image using scale-space theory for scale invariance [94] with scaling parameters selected for the LCA and RCA based on expected vessel width ranges [95]. The differential filters are optimized for rotation invariance [96]. Finally, the vesselness response of the diffused image $I'$ is given by the maximum vesselness response over all scales,

$$\begin{aligned} V = max_{\sigma } V_{\sigma } \end{aligned}$$

## Appendix 2: Computational time for different under-sampling procedures

Table 5 shows the mean and standard deviation of the computational times when applying different under-sampling methods over all images in our dataset. Unsupervised under-sampling is implemented by Matlab, while Tomek Links and Cluster Centroid are carried out with Python Imbalanced-learn library [80]. Cluster Centroid under-sampling takes 67312 times more computational time than unsupervised method, and uses 163 time more computational time than the Tomek Links under-sampling.

**Table 5:** Computational time statistics

Under-sampling methods	Computational time (seconds)
Unsupervised	0.08 ± 0.005
Tomek links	33.36 ± 8.62
Cluster centroid	5385.73 ± 207.27

## Appendix 3: Training details on DeepLabV3+

We applied a backbone of ImageNet pre-trained Resnet101, an encoder depth of 5, an encoder output stride of 16, a decoder atrous rates of (12, 24, 36), a decoder channel of 256 in the DeepLabV3+ model construction. These are the preferable parameter choices described in the original paper when training the model on images of roughly the same resolution as the ours.

## Abbreviations

AUROC: Area under the receiver operating characteristic curve
AVI: Audio video interleave
CAD: Coronary artery disease
Cath: Catheter diameter size
CNNs: Convolutional neural networks
DenseNet121: Densely connected convolutional networks, 121 layers
DICOM: Digital imaging and communications in medicine
IoU: Intersection over union
GBDT: Gradient boost decision tree
GD: Generalized dice loss
GPU: Graphics processing unit
LCA: Left coronary arteries
PCI: Percutaneous coronary intervention
QCA: Quantitative coronary angiography
RCA: Right coronary arteries
XCA: X-ray coronary angiography
